# Mitochondria and cancer

**DOI:** 10.1186/2049-3002-2-8

**Published:** 2014-06-10

**Authors:** Navdeep S Chandel

**Affiliations:** 1 Feinberg School of Medicine, Northwestern University, Chicago, USA

## Editorial

Proliferating cells such as tumor cells have increased metabolic demands that include ATP, NADPH, lipids, proteins, and nucleotides to allow for a tumor cell to divide into two daughter cells [1]. Tumor cells reprogram their cell metabolism to sustain the increased metabolic demands of cell proliferation. Historically, much attention has focused on glycolysis as the central metabolic pathway important for tumor cell metabolism, an idea that stems from the observation made in 1920s by Otto Warburg that tumor slices consume glucose at a higher rate than normal tissue slices at normal oxygen levels [2]. This high rate of aerobic glycolysis is known as the Warburg effect and can be observed in proliferating tumor cells of cancer patients by the high uptake of the glucose analogue tracer 18-fluorodeoxyglucose (FDG) detected by Positron Emission Tomography (PET) technology [3]. The basis and the advantage of the Warburg effect for proliferating cells such as cancer cells had not been fully resolved until recently.

Today, there is consensus that combination of gain of function of oncogenes, loss or tumor suppressor and aberrant activation of signaling pathways downstream of growth factor signaling induce the Warburg effect [4]. This increase in glucose metabolism through glycolysis allows the generation of glycolytic intermediates that funnel into biosynthetic pathways that support the production of NADPH, lipids, proteins and nucleotides [5]. However, the biochemists working on cancer metabolism decades ago realized that glucose metabolism alone could not fully support the de novo production of NADPH, ATP, lipids, nucleotides and proteins required for cell proliferation. Today, it is appreciated that mitochondrial metabolism is also essential for building blocks needed for cell proliferation. For example, phospholipid generation needed for de novo cell membranes in proliferating cells requires fatty acids and glycerol. The glycolytic intermediate dihydroxyacetone phosphate provides glycerol while the TCA cycle intermediate citrate transports into the cytosol where it is converted into acetyl-CoA to produce fatty acids. As glucose is the fuel for glycolysis and its subsidiary biosynthetic pathways, glutamine has emerged as a key fuel for mitochondrial metabolism [6]. A consequence of mitochondrial oxidative metabolism is the generation of reactive oxygen species (ROS) that are necessary for optimal activation of signaling pathways needed for cell proliferation [7]. Recent studies have confirmed previous work indicating that the majority of ATP in tumor cells is derived from mitochondrial oxidative phosphorylation [8].

It is important to note that mitochondria are indeed functional in most tumor cells. However, there are a fraction of tumor cells that have been shown to exhibit mitochondrial dysfunction due to loss of function mutations in the TCA cycle enzymes succinate dehydrogenase (SDH) or fumarate hydratase (FH) [9]. These cells rely on glycolysis for ATP production. However, SDH and FH null cells are still dependent on the fraction of mitochondrial TCA cycle and ETC. that is functional in these cells to generate TCA cycle intermediates such as citrate for macromolecule synthesis and ROS for signaling. Thus, mitochondria contribute to the bioenergetic, biosynthetic and signaling requirements of proliferating cancer cells. A recent bioinformatics analysis yielded enzymes in mitochondrial one-carbon metabolic pathways as the top hits of metabolic enzymes upregulated in cancer compared to normal proliferating cells [10]. Interestingly, the anti-diabetic drug metformin, which has been repurposed as an anti-cancer agent, was recently shown to inhibit mitochondrial complex I to exert its’ anti-tumorigenic properties [11]. Collectively these insights have led to the possibility of targeting mitochondria for cancer therapy. In this series on mitochondria and cancer, the diverse functions and regulators of mitochondria in controlling tumor growth are reviewed.

## Competing interests

The author declares that he has no competing interests.
